# Overall Staging Prediction for Non-Small Cell Lung Cancer (NSCLC): A Local Pilot Study with Artificial Neural Network Approach

**DOI:** 10.3390/cancers17030523

**Published:** 2025-02-04

**Authors:** Eva Y. W. Cheung, Virginia H. Y. Kwong, Kaby C. F. Ng, Matthias K. Y. Lui, Vincent T. W. Li, Ryan S. T. Lee, William K. P. Ham, Ellie S. M. Chu

**Affiliations:** 1 Department of Diagnostic Radiology, School of Clinical Medicine, LKS Faculty of Medicine, University of Hong Kong, Hong Kong; 2 Department of Clinical Oncology, Prince of Wales Hospital, Hong Kong; 3 School of Medical and Health Sciences, Tung Wah College, Hong Kong

**Keywords:** radiomics, non-small cell lung cancer NSCLC, neural network, overall staging, CT image, artificial intelligence, feed-forward neural network, pattern recognition neural network, lung cancer, NSCLC, artificial neural network

## Abstract

Background: Non-small cell lung cancer (NSCLC) has been the most common cancer globally in the recent decade. CT is the most common imaging modality for the initial diagnosis of NSCLC. The gold standard for definitive diagnosis is the histological evaluation of a biopsy or surgical sample, which usually requires a long processing time for the confirmation of diagnosis. This study aims to develop artificial intelligence models to predict overall staging based on patient demographics and radiomics retrieved from the initial CT images, so as to prioritize later-stage patients for histology evaluation to facilitate cancer diagnosis. Method: Two cohorts of NSCLC patient datasets were utilized for this study. The NSCLC-radiomics dataset from The Cancer Imaging Archive (TCIA) was divided into 70% for the training group and 30% for the internal testing group. Another cohort from a local hospital was collected for the an external testing group. Patient demographics and 107 radiomic features were retrieved from the gross tumor volume delineated by clinical oncologists on CT images. Artificial neural networks were used to build models for NSCLC overall staging (stage I, II, or III) prediction. Four traditional classifiers were also adopted to build models for comparison. Result: The proposed feed-forward neural network (FFNN) model showed good performance in predicting overall staging with an accuracy of 88.84%, 76.67%, and 74.52% in overall accuracies in validation, internal cohort testing, and external cohort testing, respectively. The sensitivity and specificity are balanced in all the stages, with average precision and F1 score in each of the stages. Conclusion: The FFNN demonstrated good performance in overall staging prediction for NSCLC patients. It has the benefit of predicting multiple overall stages in a single model. The software required and the proposed model are simple. It can be operated on a general-purpose computer in the radiology department. The application will eventually be used as a prediction tool to prioritize the biopsy or surgery sample for histological analysis and molecular investigation, thus shortening the time for diagnosis by pathologists, which supports the triage of patients for further testing.

## Simple Summary

CT is a commonly available imaging modality for the preliminary diagnosis and staging of non-small cell lung cancer (NSCLC). While a multidisciplinary team of experts is required to confirm it, this study aimed at building an AI model for overall staging prediction for NSCLC patients based on CT images. The feed-forward neural network (FFNN) AI model achieved overall accuracies of 88.84%, 76.67%, and 74.52% in model training, and testing using an internal cohort and external cohort, respectively. It also achieved the highest accuracy in characterizing all stages I, II, and III, with balanced sensitivity and specificity. The FFNN model can predict multiple stages in one model, which can be used as a preliminary staging tool to prioritize patients for the biopsy or surgery sample collection, so as to improve the pathway of NSCLC diagnosis confirmation.

## 1. Introduction

In 2020, lung cancer was the second most commonly diagnosed cancer (11.4% of total cancers, with 2.2 million cases) and the leading cause of cancer deaths (18% of all cancers, with 1.8 million cases) globally [1]. In Hong Kong, it has the highest incidence among all cancers and has been the leading cause of cancer deaths in the past decade. With respect to the cancer registry, 85% of primary lung cancers are non small cell lung cancers (NSCLC) [2]. The overall staging is crucial in determining the extent of the diseases, guiding treatment decisions, predicting prognosis, and facilitating communication among healthcare professionals.

The process of staging is a critical component in the management and treatment of lung cancer, as it helps to determine the extent of the disease, guides the treatment decisions, and provides prognostic information. Routine lung cancer staging is a complex process. It involves a systematic evaluation of the tumor’s histology, size, location, and whether it has spread to other parts of the body. The process requires a multidisciplinary team of experts, including radiologists, pathologists, oncologists, and other specialists. CT is a popular imaging modality, which has been employed in lung cancer staging conducted worldwide [3,4,5]. The tumor volume, the lungs, lymph nodes, as well as nearby organs, are presented in one CT image set. This allows clinicians to have a full view of cancer development and the extent of the disease [6,7].

The TNM staging is the most widely used cancer staging system, where T refers to the size and extent of the primary tumor, N refers to the number of lymph nodes or whether certain lymph node stations are involved, and M refers to whether the cancer has metastasized [8]. Once the TNM stages have been determined, they can be combined to assign an overall stage to the disease to describe the development of cancer. This staging system is developed by the American Joint Committee on Cancer (AJCC) and the Union for International Cancer Control (UICC) [9]. It assigns stages from I to IV, with stage I indicating localized lung cancer and stage IV indicating advanced or metastatic lung cancer.

The final diagnosis requires confirmation from pathological examination on biopsy or surgical samples, including cancer cell morphology analysis on hematoxylin and eosin stain (H&E stain) and specific immuno-staining. It usually takes a few weeks to a month for results. The overall staging prediction from CT images and clinical data can prioritize the diagnostic process, by arranging earlier pathology examination for NSCLC patients, to shorten the time for diagnosis.

Radiomics is the use of mathematical algorithms to extract data from medical imaging [10]. It provides quantitative image features that are associated with clinical, pathological, and molecular characteristics for diagnosing and staging lung cancer. It also allows researchers to define features from images related to lung tumors. Compared to the conventional method using clinical parameters and traditional radiology, previous studies demonstrated that radiomics can be a more nuanced method that integrates radiology and statistics [11,12,13]. The application of radiomics, together with deep learning, which is applied to develop association and representation levels of sample data for model building, made a meaningful contribution to cancer staging [14]. Within TNM staging, tumor size and shape irregularity are crucial to determine the T stage. They can be retrieved from the first-order statistics, two-dimensional, and three-dimensional shape features in radiomic features in CT images [15]. In addition to CT radiomics, recent researchers suggested that sex and age at diagnosis are predictors for lung cancer staging, where females diagnosed at a younger age (under age 50) had a higher proportion diagnosed with stages I-II [16]. Another study conducted by Paakkola et al., 2023 suggested that older patients are usually diagnosed at a later stage of the disease [17].

Artificial intelligence (AI) and machine learning have been employed in various aspects of oncology, including cancer diagnosis [18,19], prognosis prediction [20,21,22], metastases prediction [23], and recurrence prediction [24]. Most of the machine-learning algorithms used in oncology are based on binary classification that can either provide positive or negative outcomes, which limits their applications in clinical situations. In recent years, the development of artificial neural networks (NN) has gained researchers’ attention as their architecture simulates the human brain, with a training process that mimics the human learning mechanisms. Compared to binary classification machine learning algorithms, the artificial neural network offers the benefits of allowing multiple-class classification [25] and, therefore, could be an ideal model for differentiating various stages of NSCLC.

In this study, we aimed to develop an overall staging prediction system by combining patients’ demographics and CT radiomics using artificial intelligence (AI) neural networks. It is expected that the neural networks proposed to attain high accuracy in predicting the overall staging based on the radiomics retrieved from CT images. The application of the proposed neural network could be an aid to speed up the diagnostic pathway for cancer confirmation and allow early planning of treatment for NSCLC patients.

## 2. Materials and Methods

### 2.1. Patient Dataset

There were two independent datasets used in this study. The first dataset was NSCLC-Radiomics, which was obtained from The Cancer Imaging Archive (TCIA) [26]. This dataset (TCIA dataset) comprises 422 NSCLC patients at three stages of the disease, i.e., Stage I, II, and III. This dataset comprises CT images, a radiotherapy structure set, segmentations in DICOM format, and demographic data. Patient demographic data, including sex, age at diagnosis, survival days after confirmed diagnosis, TNM staging, and overall staging (i.e., pathological post-surgical stage), were collected. The disease was staged using the 8th Edition of the TNM system published by the American Joint Committee on Cancer (AJCC-TMN) [27]. The organ segmentation sets contain 3D primary gross tumor volume (GTV) and nearby organs at risk, including the lung, esophagus, and heart. These targets and organs were delineated and verified by experienced radiation oncologists in this project [26].

Another independent dataset was retrieved locally from the Clinical Oncology Department at Prince of Wales Hospital (PWH) in Hong Kong. Patients who suffered from Stage I, II, or III NSCLC and received treatment in PWH with radical intent during 2014–2018 were included in this study. Patients who suffered from small cell lung cancer, who suffered from stage IV NSCLC, or who received palliative treatment were excluded from this study. This dataset (PWH dataset) also comprises CT images, a radiotherapy structure set, segmentations in DICOM format, and demographic data. Patient demographics, including sex, age at diagnosis, survival days after confirmed diagnosis, TNM staging, and overall staging, were collected. For patients who were diagnosed in 2014–2017, the disease was originally staged using the 7th Edition of the TNM system published by the American Joint Committee on Cancer (AJCC-TMN) [27]. For those who were diagnosed in 2018, the disease was staged using the 8th Edition of the TNM system published by the American Joint Committee on Cancer (AJCC-TMN) [9,28]. To unify the disease staging, all patients were reviewed, restaged, and assigned an overall stage using the 8th Edition of the TNM system published by the American Joint Committee on Cancer (AJCC-TMN) [9,28]. The staging, target volume, and organs were performed by oncologists of PWH.

### 2.2. Ethics Approval

Formal consent from all patients who participated in the TCIA NSCLC-radiomics study was collected through the participating institutions during data collection. For the PWH dataset, ethics approval was obtained from the Joint Chinese University of Hong Kong, New Territories East Cluster Clinical Research Ethics Committee with CREC reference number 2021.628.

### 2.3. Features for Model Building

#### 2.3.1. Patient Demographics

Patient’s sex and age at diagnosis were collected from the clinical database and were used as input features for model building.

#### 2.3.2. Radiomics Extraction from CT Images

**Figure 1:**
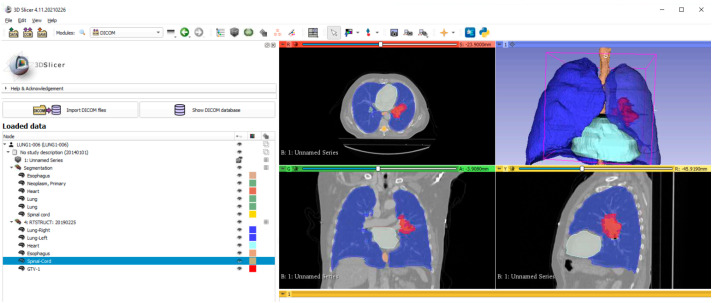
The radiomic features extraction from 3D slicers.

One hundred and seven radiomic features were extracted from the gross tumor volume (GTV) in the organ structure set (red structure in Figure 1). It was performed using 3D slicer software (The Slicer Community; V.4.11.20210226) equipped with the PyRadiomics extension (Computational Imaging and Bioinformatics Lab, Harvard Medical School, Boston, MA, USA) [29]. The definition of radiomic features was subdivided into eight categories [30]. All image features are in compliance with the definition described by the Image Biomarker Standardization Initiative [31]. Details are listed in Table 1.

### 2.4. Study Workflow

**Figure 2:**
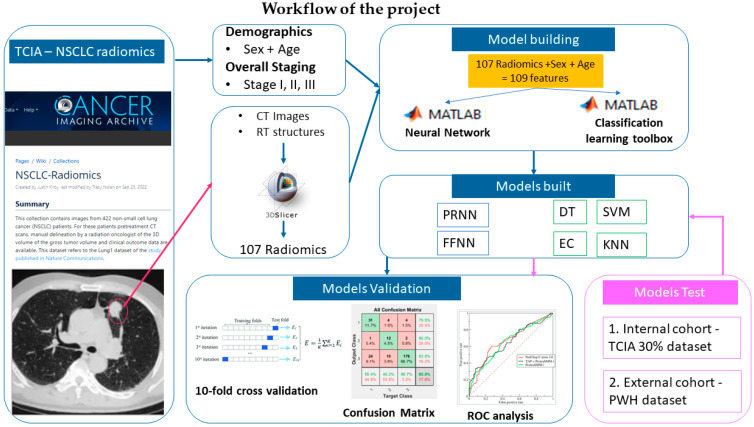
The workflow of the study.

The study consisted of two parts. The first part aimed to build the models for NSCLC overall staging prediction using the TCIA dataset (blue in Figure 2). The second part aimed to test the models built in the first part with internal and external data (pink in Figure 2).

The TCIA dataset was split into three independent groups, with 70% for the training group and 30% for the testing group at random. The validation result and the testing result were reported as TCIA-Validate and TCIA-test, respectively. The PWH dataset was used as an external cohort for model deployment. The result was reported as a PWH-test.

### 2.5. Artificial Neural Network Building

**Figure 3:**
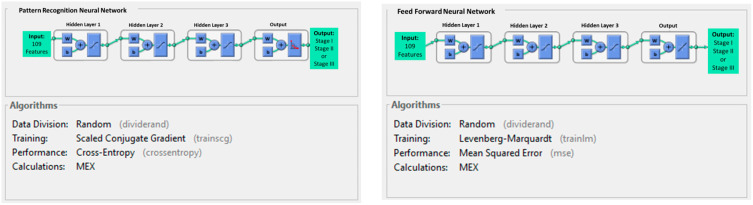
Details of the PRNN and FFNN models. W represents the weight of the neuron. b represents the bias of the neuron.

The pattern recognition neural network (PRNN) and feed-forward neural network (FFNN) algorithms were used to build the overall staging prediction model. Both algorithms were run on the MATLAB^®^ 2021a platform. During model building, the training processes were optimized using the hyper-parameter optimization algorithm. The maximum epoch was set to 50, and the training time was not limited. The three-layered PRNN was trained using a scaled conjugated gradient algorithm with cross-entropy loss to adjust model weights during training. The three-layered FFNN was trained using the Levenberg–Marquardt algorithm with a mean squared error performance function and a regularization value of 0.01. The model was trained with early stopping-based optimization, i.e., the model training was stopped when the performance function and a regularization value of 0.01 were achieved. Details of the PRNN and FFNN model building are listed in Figure 3.

The complexity of the network, including the number of neurons per hidden layer and the number of hidden layers, was adjusted. Based on the hyper-parameter optimization algorithm, the best-performing model of each algorithm was reported.

To facilitate the on-site preliminary diagnosis, the neural network was established based on a balance between accuracy, processing time, and computer performance. In this study, the neural network models were designed based on the following criteria: the overall accuracy is over 75% after cross-validation; the processing time for the neural network is within 1 min; and the neural network is light-weighted (i.e., not require GPU or high demand in memory), which can be operated on a general-purpose workstation. In the end, the PRNN model and FFNN model were built.

Traditional classifiers, including decision tree (DT), support vector machine (SVM), ensemble classifier (EC), and k-nearest neighbors (KNN) in the Matlab Classification Learner Toolbox, were utilized to build an additional 4 models for comparison. The Bayesian optimization algorithm was employed as the hyper-parameter tuning, with a maximum of 30 iterations and no training time limit in DT, SVM, EC, and KNN model building. The setting was applied to improve the stability and accuracy of the models [32]. In the end, the DT model, SVM model, EC model, and KNN model were built.

### 2.6. Cross Validation

To improve the generalization of the neural networks, and maximize the use of all patient data, the 10-fold cross-validation was employed to measure the accuracy of the neural network. The dataset was divided into ten groups with an equal number of samples. During the first training, nine groups of data were used, and the remaining group was used for testing. In the consecutive trainings, another nine groups were used as training data and the rest were used as testing data. The process continued 10 times with no repeated use of training data. The average performance of the 10-time training was computed and reported [33].

### 2.7. Data Analysis

The performance of the 2 neural network models and 4 traditional classifier models was assessed by the overall accuracy, confusion matrix with sensitivity and specificity, and receiver operating characteristic (ROC) curve. The results were listed as TCIA-validate.

### 2.8. Models Testing

The performance of the 2 neural network models and 4 traditional classifier models were further tested by the internal cohort of data, i.e., the TCIA test dataset, and the external cohort of dataset, i.e., the PWH dataset. The overall accuracy, confusion matrix with sensitivity and specificity, and receiver operating characteristic (ROC) curve were reported as TCIA-test and PWH, respectively. The workflow was illustrated in the pink park in Figure 2.

## 3. Results

### 3.1. Patient Demographics of Two Datasets

#### 3.1.1. TCIA Dataset

This dataset comprises 422 NSCLC patients at three stages of the disease, i.e., Stage I, II, and III. However, there was one patient with incomplete CT images and 22 patients without age information. Thus, 23 patients were excluded from this study. As a result, a total of 399 patients were included in this study. All 399 patients were randomly split into two independent groups, TCIA-training with 70% (279 patients), and TCIA-testing with 30% (120 patients) for training and testing groups, respectively.

#### 3.1.2. PWH Dataset

There were 311 patients included in this study. There was one patient with incomplete CT images. As a result, 310 patients were included in this study. Details of both datasets are illustrated in Table 2.

### 3.2. Performance of Artificial Neural Networks, i.e., PRNN and FFNN in Overall Staging Prediction

Among the two artificial neural network models, the FFNN model performed better than the PRNN. The FFNN model achieved 88.84%, 76.67%, and 74.52% in overall accuracy for model training and testing using internal cohort and external cohort, respectively. It also attained the highest accuracy in characterizing all stages I, II, and III, with balanced sensitivity and specificity, precision and F1 score. The results are listed in Table 3.

### 3.3. Performance of Artificial Neural Networks When Compared to Traditional Classifiers in Overall Staging Prediction

Overall, in all models built by the classifiers, the overall accuracies ranged from 66.31 to 70.9% in TCIA-validation, and from 62.9–to 70.8% in the testing dataset. For individual stage classification, SVM achieved the highest accuracies in all stages. However, the sensitivity in stage II was 0, indicating that SVM is not able to classify stage 2 effectively. Details of the results are listed in Table 4. 

### 3.4. Important Features to Build the Neural Network Models

In both neural networks, the top ten most important features that contributed to building the overall staging prediction models were large dependence high gray level emphasis, large area high gray level emphasis, cluster prominence, variance, range, voxel volume, mesh volume, long run high gray level emphasis, surface area, and complexity. Details and their relative importance are listed in Table 5.

## 4. Discussion

### 4.1. The Value of Artificial Neural Network Models for NSCLC Overall Staging Prediction

There are several studies conducted using AI for NSCLC staging. Masood and his colleagues used a convolution neural network to classify tumor staging (i.e., T stage only). They achieved accuracies of 78% to 96% in T1 to T4 [34]. However, the T stage of the tumor is just part of the overall staging. It required further information, including lymph node involvement and details of metastases to form the overall staging, which is clinically more important to govern the treatment regimen. Other studies used a single dataset for lung cancer staging detection and classifications [35,36], while the performance of those models was yet to be validated through an independent dataset.

In the current study, an artificial neural network was employed to build the NSCLC overall staging prediction model. NSCLC patients may suffer from stage I to stage III. This model allows multiple-class classification in one neural network for the analysis of a complex situation. In addition, a local independent cohort of patients was used for model validation. This improves the model’s generalizability and suitability for local clinical applications.

In real-world clinical environments, patients are classified in various overall stages based on the clinical criteria of protocol; class imbalance is common. Both TCIA and PWH datasets used in the current study demonstrated class imbalance, with fewer patients classified in stage II compared to stages I and III. Due to the imbalanced samples, machine learning models may skew toward stage I and III [37]. If the model is assessed by overall accuracies only, good accuracy may result from the averaging effects of good sensitivity and poor specificity (or vice versa). In this study, a comprehensive analysis method is employed to ensure the model prediction is accurately assessed in all three classes. A full confusion matrix, including sensitivity, specificity, precision, and F1 score, was used to analyze the models in this study. The F1 score elaborates on the model’s class-wise performance rather than an overall performance. It combines precision and recall by using their harmonic mean. Therefore, maximizing the F1 score indicates maximizing both precision and recall. It is particularly useful in analyzing models with imbalanced classes [38]. Our study results showed that FFNN had good prediction accuracies for all three stages, with balanced sensitivity and specificity, and averaged precision and F1 score. On the other hand, the precision and F1 scores were poor in stage II classification in all traditional classifiers, even though the accuracies were over 80% in all validation and testing. The significant improvement of prediction capabilities in NN over traditional classifiers may be due to the calculation algorithms employed and their architecture.

For datasets that have imbalanced classes, e.g., datasets for credit card fault transaction detection models in financial sectors, the fault transactions are much less than the normal ones. The synthetic minority over-sampling technique (SMOTE) has been employed to enhance the model performance [39,40,41]. It focused on extracting the characteristics of the minority group, and generating more samples for the minority group synthetically [42]. The performance of the models was improved by oversampling the minority class and undersampling the majority class. However, in the clinical setting, where each patient presents unique characteristics, SMOTE application remains controversial. A previous study demonstrated that using SMOTE to introduce 62 patients in the minority group did improve the model’s classification performance, though the major features extracted for model building deviated from clinically validated contributors [43]. In the current study, other than using SMOTE to oversample the minority group, high-level neural network models were employed to improve the overall staging prediction performance. The features extracted were analyzed, and they aligned with the diagnostic criteria of NSCLC. Thus, the model developed would be reliable and clinically compatible.

Various neural network models have been developed to mimic human decision-making logic, including PRNN and FFNN. They have similar architecture, which is composed of four layers (three hidden layers and one output layer). The NN analyzes the input data and develops the association with the output by adjusting the weights and biases to input data between nodes for multiple-class classification [25]. The choice of the number of hidden layers depends on the complexity of the task, feature hierarchy, and expressive power. Heaton suggested that zero hidden layers are needed if the data are linear with binary output. Two hidden layers are required to represent arbitrary decisions, and more than two hidden layers can learn complex representations. However, more hidden layers may induce model overfitting [44]. Two hidden layers were employed initially for model building, yet the accuracy was unsatisfactory. Thus, three hidden layers were used in both PRNN and FFNN models in this study. Our results demonstrated that both NN models achieved good accuracy without overfitting. The main difference between PRNN and FFNN is the calculation algorithm, where PRNN used a scaled conjugated gradient that treated the step size as a function of quadratic approximation of the error function [45]; while the FFNN used the Levenberg–Marquardt algorithm, which considers both linear and non-linear characteristics of input parameters [46]. The advantage of the scaled conjugated gradient calculation algorithm used in PRNN has relatively modest memory requirements, and it performs well in a wide variety of applications with a large number of weights. However, the Levenberg–Marquardt algorithm used in FFNN demonstrated the fastest convergence, which is significant when applied to a network for accurate training [47].

### 4.2. The Important Features to Build the Neural Network Models for NSCLC Overall Staging Prediction

#### 4.2.1. The 3D Shape Features—Voxel Volume, Mesh Volume, and Surface Area

With respect to the eighth edition of the TNM system, T refers to the tumor size and extent of the primary tumor [28]. The bigger the tumor, the higher the overall stage for NSCLC patients. The voxel volume is the representation of the approximated volume of the GTV, obtained by multiplying the number of voxels within the GTV by the volume of a single voxel. The mesh volume is the actual volume of GTV calculated from the triangle mesh of GTV. The surface area is proportional to the radius of the tumor [48]. These three features were important in developing the model that aligns with the staging criteria of the TNM system.

#### 4.2.2. The First-Order Statistics—Variance and Range

Variance is the mean of the squared distances of each intensity value from the mean value. The range is the span of gray values within the GTV. The larger the variance and range of gray intensity in GTV, the more heterogeneous it is, which is more common in higher-grade tumors. Tumor heterogeneity has been used as an important tumor prognostic factor [49], which has a similar effect to the TNM system.

#### 4.2.3. Features Related to High Gray Level Emphasis

High gray level emphasis measures the distribution of the higher gray level values. The large dependence high gray level emphasis measures the joint distribution of large dependence with higher gray value levels. Large area high gray level emphasis measures the proportion in the image of the joint distribution of larger size zones with high gray level values. Long run high gray level emphasis indicates where long run lengths (i.e., consecutive sequence of voxel) with the same high gray levels voxels are located [50]. The higher the high gray level emphasis value, the greater the concentration of high gray level values in the GTV [30]. Previous studies suggested that the larger the dependence, the larger the area and the longer the high gray level emphasis showed a positive correlation with prognostication in pancreatic neuroendocrine tumors [51] and gastrointestinal stromal tumors [52]. Our study had similar results where the large dependence high gray level emphasis, the large area high gray level emphasis and long run high gray level emphasis are important features for predicting the various stages for NSCLC patients.

#### 4.2.4. Cluster Prominence and Complexity

Cluster Prominence measures the skewness and asymmetry of gray level co-occurrence matrix. A higher value indicates greater asymmetry about the mean [30]. A previous study demonstrated that the location of high cluster prominence indicated calcification in mammograms [53]. Complexity within the GTV indicates rapid changes in gray level intensity [30]. The heterogeneity characteristics of GTV demonstrated a sharp fall-off of voxel intensity which may contribute to the high complexity [54]. Our results showed that both cluster prominence and complexity are important features in the overall staging prediction of NSCLC. These may be due to calcification or necrotic regions, which demonstrate rapid changes in gray level intensity. Further investigation may be required to correlate the application of both features within the GTV of NSCLC.

### 4.3. Potential Clinical Application of the Proposed Neural Networks

The proposed FFNN model developed in this study can be installed in a CT imaging workstation as software within Matlab. It is lightweight and compatible with the CT viewing workstation. After the CT image is acquired, gross tumor volume is delineated on the CT image automatically by segmentation software and verified by clinical staff. Radiomics can be retrieved as a text file and act as inputs for the neural networks. Clinical staff can click the FFNN model software, load the radiomics, and the overall staging can be predicted using the model built in this study automatically. The resultant overall staging can be used to establish preliminary staging, which aids clinical staff in triaging patients with reference to their medical condition. The preliminary staging provides additional information for radiologists to determine whether further testing is required. Priority can be given to those patients with urgent conditions for biopsy, ultimately speeding up laboratory testing in order to facilitate the diagnosis by pathologists. The waiting time for diagnosis confirmation for NSCLC patients can be minimized, which may potentially improve their overall prognosis and quality of life.

### 4.4. Main Finding of Study

In this study, we utilized the radiomics retrieved from CT images and patient demographics to build neural networks for overall staging prediction for NSCLC patients. The performance of the models built was evaluated through sensitivity, specificity, ROC curve, and overall accuracy. The proposed FFNN model demonstrated the best distinguishing ability and achieved very good performance in overall staging prediction in both internal and external cohorts of NSCLC patients.

### 4.5. Study Limitations and Further Studies

In this study, only a single cohort of NSCLC patients from the TCIA database was used for model training. The sample size was relatively small, with imbalanced samples in various stages. Further studies are suggested by integrating other cohorts of NSCLC patients, to increase the sample size for comprehensive testing of the proposed networks.

In addition, radiomics from CT images and patient demographics were used as inputs to develop the neural network for overall staging prediction. The model’s performance can be improved by integrating image features from other imaging modalities. For cancer diagnosis, PET/CT with 18F-FDG radiopharmaceutical and MRI are commonly used. The radiomic features retrieved from PET/CT and MRI may aid in improving the overall accuracy of the proposed neural networks [55,56]. Furthermore, feature-based analysis of hematoxylin and eosin-stained slides is the gold standard of cancer diagnosis. Including histopatholomics as inputs into the neural network building may improve the accuracy of cancer staging prediction [57]. Additionally, other clinical parameters, such as biomarkers, including epidermal growth factor receptor, and the programmed death-1 (PD-1) ligand 1 (PD-L1) expression from histological and molecular analyses for NSCLC can be incorporated as input features for model modification, potentially improving the prediction capabilities with relevant parameters in other perspectives [58,59].

## 5. Conclusions

This study focused on building an artificial neural network for overall staging prediction for NSCLC patients. The FFNN model yielded good accuracy for overall staging prediction, with 88.84%, 76.67%, and 74.52% in overall accuracies in validation, internal cohort testing, and external cohort testing, respectively; with balanced sensitivity and specificity, as well as average precision and F1 score in each of the stages I, II, and III, respectively. The FFNN has the benefit of predicting multiple stages in one model. The software required and the proposed networks are simple. It can be operated on a general-purpose computer in the radiology department. The application may be used as a preliminary prediction tool to prioritize patients for biopsy or surgery sample collection, so as to conduct histological analysis and molecular investigation. This will shorten the time for diagnosis by pathologists, which supports the triage of patients for further testing.

## Figures and Tables

**Table 1:** The 107 radiomic features in eight categories.

Radiomics Features	No of Features
First-order statistics	14
2D shaped based features	9
3D shaped based features	13
Gray level co-occurrence matrix (GLCM)	22
Gray level run length matrix (GLRLM)	16
Gray level size zone matrix (GLSZM)	16
Gray level dependence matrix (GLDM)	12
Neighboring gray tone difference matrix (NGTDM)	5
**Total**	**107**

**Table 2:** Patient demographics of TCIA and PWH datasets.

			Training	Testing— Internal Cohort	Testing— External Cohort
	TCIA Dataset 100%	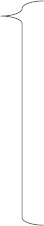	TCIA Dataset 70%	TCIA Dataset 30%	PWH Dataset
Age	33.68–91.70 ±10.11				37–93 ± 10.96
Sex (M:F)	274:125				211:99
Overall Stage					
I	84		58	26	68
II	37		26	11	53
III	278		195	83	189
Total	399		279	120	310

**Table 3:** Results of PRNN and FFNN. TCIA-Validated was the result when building the model using patients from TCIA 70% dataset; TCIA-Test was the result when testing the model by patients from TCIA 30% dataset; PWH was the result when testing the model by patients from PWH dataset. The blue fonts highlight results over 70%.

		Overall			STAGE 1					STAGE 2					STAGE 3		
		Accuracy	Accuracy	Sensitivity	Specificity	Precision	F1 score	Accuracy	Sensitivity	Specificity	Precision	F1 score	Accuracy	Sensitivity	Specificity	Precision	F1 score
PRNN	**TCIA** **Validate**	76.89%	85.66%	72.97%	87.85%	50.94%	60.00%	90.04%	41.67%	92.47%	21.74%	28.57%	78.09%	79.70%	71.43%	92.00%	85.41%
	**TCIA Test**	61.67%	74.17%	35.29%	80.58%	23.08%	27.91%	84.17%	27.78%	94.12%	45.45%	34.48%	65.00%	74.12%	42.86%	75.90%	75.00%
	**PWH**	56.77%	69.68%	29.69%	80.08%	27.94%	28.79%	82.90%	50.00%	85.42%	20.75%	29.33%	60.97%	65.18%	50.00%	77.25%	70.70%
FFNN	**TCIA** **Validate**	88.84%	90.44%	76.36%	94.39%	79.25%	77.78%	96.02%	84.21%	96.98%	69.57%	76.19%	91.24%	93.22%	86.49%	94.29%	93.75%
	**TCIA Test**	76.67%	85.83%	69.57%	89.69%	61.54%	65.31%	88.33%	42.11%	97.03%	72.73%	53.33%	79.17%	87.18%	64.29%	81.93%	84.47%
	**PWH**	74.52%	82.90%	61.19%	88.89%	60.29%	60.74%	86.45%	61.22%	91.19%	56.60%	58.82%	79.68%	82.47%	75.00%	84.66%	83.55%

**Table 4:** Results of SVM, DT, KNN, and EC. TCIA-Validated was the result when building the model using patients from TCIA 70% dataset; TCIA-Test was the result when testing the model by patients from TCIA 30% dataset; PWH was the result when testing the model by patients from PWH dataset. The blue fonts highlight results over 70%.

		Overall			STAGE 1					STAGE 2					STAGE 3		
		Accuracy	Accuracy	Sensitivity	Specificity	Precision	F1 score	Accuracy	Sensitivity	Specificity	Precision	F1 score	Accuracy	Sensitivity	Specificity	Precision	F1 score
**SVM**	**TCIA** **Validate**	70.61%	80.65%	59.09%	82.49%	22.41%	32.50%	89.96%	0.00%	90.61%	0.00%	0.00%	70.61%	72.16%	54.17%	94.36%	81.78%
	**TCIA Test**	70.83%	80.00%	66.67%	80.70%	15.38%	25.00%	90.83%	0.00%	90.83%	0.00%	0.00%	70.83%	71.05%	66.67%	97.59%	82.23%
	**PWH**	64.84%	77.74%	48.72%	81.92%	27.94%	35.51%	82.90%	0.00%	82.90%	0.00%	0.00%	69.03%	67.16%	82.05%	96.30%	79.13%
**DT**	**TCIA** **Validate**	70.97%	79.57%	51.85%	82.54%	24.14%	32.94%	90.32%	33.33%	90.94%	3.85%	6.90%	72.04%	73.49%	60.00%	93.85%	82.43%
	**TCIA Test**	66.67%	75.83%	33.33%	79.28%	11.54%	17.14%	90.83%	50.00%	91.53%	9.09%	15.38%	66.67%	69.72%	36.36%	91.57%	79.17%
	**PWH**	63.19%	76.87%	44.64%	84.06%	38.46%	41.32%	79.48%	30.77%	83.99%	15.09%	20.25%	70.03%	71.56%	65.85%	85.19%	77.78%
**KNN**	**TCIA** **Validate**	68.46%	77.06%	42.86%	83.12%	31.03%	36.00%	90.32%	40.00%	91.24%	7.69%	12.90%	69.53%	73.71%	48.94%	87.69%	80.09%
	**TCIA Test**	69.17%	79.17%	60.00%	80.00%	11.54%	19.35%	89.17%	25.00%	91.38%	9.09%	13.33%	70.00%	71.17%	55.56%	95.18%	81.44%
	**PWH**	63.55%	77.10%	46.81%	82.51%	32.35%	38.26%	82.90%	50.00%	83.12%	1.89%	3.64%	67.10%	66.67%	69.39%	92.06%	77.33%
**EC**	**TCIA** **Validate**	66.31%	77.78%	44.44%	82.72%	27.59%	34.04%	87.81%	0.00%	90.41%	0.00%	0.00%	67.03%	71.91%	40.91%	86.67%	78.60%
	**TCIA Test**	65.00%	75.83%	28.57%	78.76%	7.69%	12.12%	86.67%	0.00%	90.43%	0.00%	0.00%	67.50%	70.37%	41.67%	91.57%	79.58%
	**PWH**	62.90%	77.42%	47.83%	82.58%	32.35%	38.60%	80.97%	12.50%	82.78%	1.89%	3.28%	67.42%	67.19%	68.52%	91.01%	77.30%

**Table 5:** Important features to build the neural networks.

Features	Category	Importance
Large dependence high gray level emphasis	GLDM	1744
Large area high gray level emphasis	GLSZM	1488
Cluster prominence	GLCM	994
Variance	First order	925
Range	First order	646
Voxel volume	3D shape feature	500
Mesh volume	3D shape feature	472
Long run high gray level emphasis	GLRLM	434
Surface Area	3D shape feature	390
Complexity	NGTDM	323

## Data Availability

Publicly available dataset was used in this study. The TCIA dataset can be obtained at following website: https://www.cancerimagingarchive.net/collection/nsclc-radiomics/, DOI:10.7937/K9/TCIA.2015.PF0M9REI, (accessed on 22 January 2024). Data used in preparation of this article were obtained from the Cancer Imaging Archive (TCIA) database (https://wiki.cancerimagingarchive.net/display/Public/Wiki) (accessed on 22 January 2024). The PWH dataset are not publicly available for patient privacy protection purposes.
